# Anterior and Inferior T-Wave Inversion: A Diagnostic Challenge and the T_1_T_2_T_3_/_III_ Pattern

**DOI:** 10.1016/j.jaccas.2026.109446

**Published:** 2026-07-25

**Authors:** Jesmar Alejandro Ramonis Quintero, Daesub Chung Kwon

**Affiliations:** Department of Cardiology, University Hospital of Gran Canaria Dr. Negrín, Las Palmas de Gran Canaria, Spain

**Keywords:** acute coronary syndrome, electrocardiography, pulmonary embolism, right ventricular strain, T-wave inversion

## Abstract

**Case Summary:**

A 73-year-old man presented with progressive dyspnea, chest pain, and elevated cardiac biomarkers. Electrocardiography showed T-wave inversion in the anterior and inferior leads, maximal in V1-V3 and III. Although often suggestive of acute coronary syndrome, this distribution reflected right ventricular involvement. We propose the T_1_T_2_T_3_/_III_ pattern, a descriptive mnemonic inspired by the classic S_I_Q_III_T_III_ pattern, for this characteristic distribution of repolarization abnormalities.

**Take-Home Message:**

Recognition of the T_1_T_2_T_3_/_III_ pattern may facilitate early identification of pulmonary embolism with right ventricular involvement and help distinguish it from coronary ischemia.

## Graphical Abstract

**Figure:**
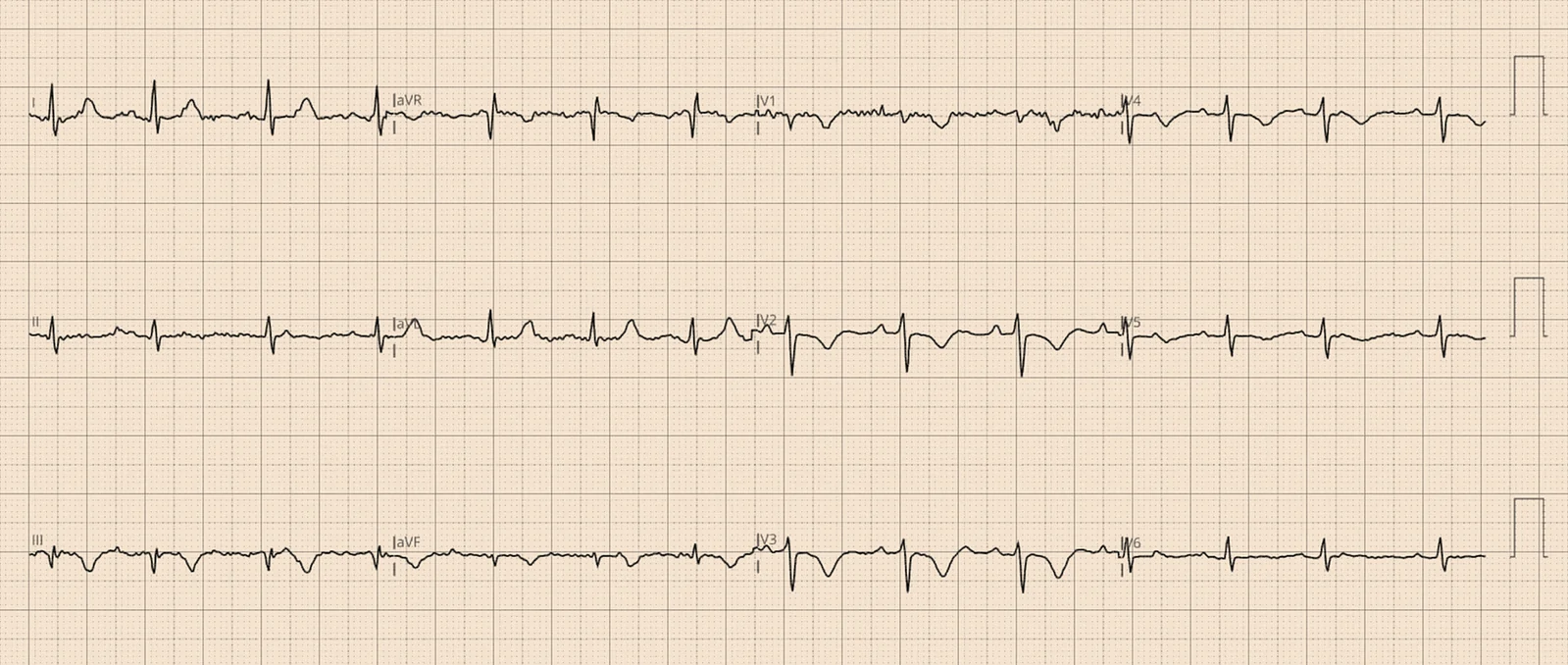

## Case

**Figure 1:**
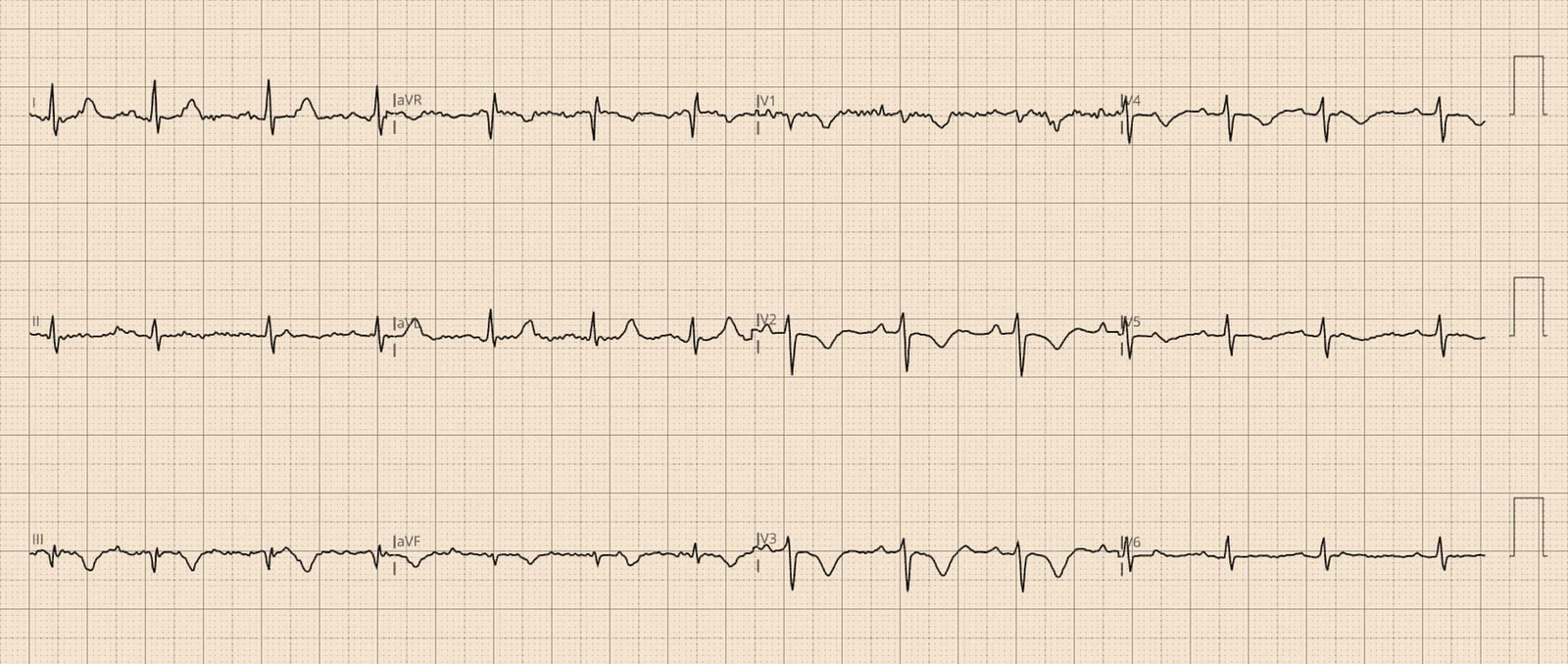
Initial 12-Lead Electrocardiogram at Presentation.

A 73-year-old man with a history of hypertension, dyslipidemia, and a recent ischemic stroke (4 months earlier) presented with a 5-day history of progressive dyspnea and intermittent oppressive chest pain. On examination, he was mildly tachypneic, with otherwise unremarkable findings. Laboratory test results revealed an elevated high-sensitivity cardiac troponin T level (110 ng/L; normal <14 ng/L) and an N-terminal pro–B-type natriuretic peptide level (6,000 ng/L). A 12-lead electrocardiogram was obtained (Figure 1).

**Take-Home Message**

• Not all anterior T-wave inversions are coronary; the mnemonic T_1_T_2_T_3_/_III_ pattern, characterized by predominant inversion in V1-V3 and III, may facilitate early recognition of pulmonary embolism with right ventricular involvement and help avoid misdiagnosis as acute coronary syndrome.

Which is the most likely diagnosis?

1. Wellens syndrome
2. Acute pulmonary embolism (PE) with right ventricular strain
3. Takotsubo cardiomyopathy
4. Apical hypertrophic cardiomyopathy

## Explanation

The electrocardiogram (Figure 1) shows sinus rhythm at approximately 75 beats/min, with a normal axis and narrow QRS complexes. There is T-wave inversion in the anterior (V1-V5) and inferior leads (III-aVF).

**Figure 2:**
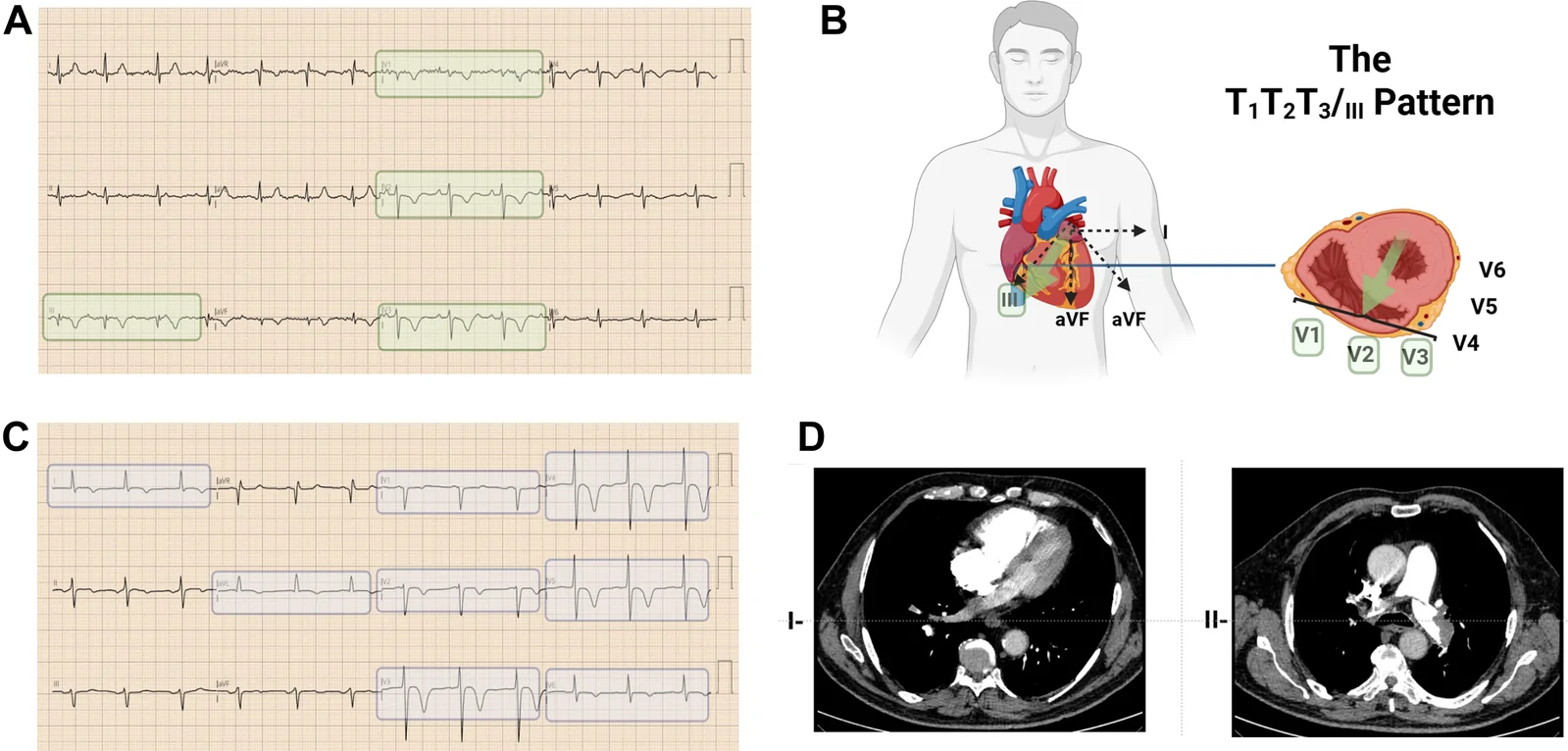
The T_1_T_2_T_3_/_III_ Pattern: Electrocardiographic Distribution, Differential Diagnosis, and Imaging Correlation (A) T-wave inversion is most pronounced in leads V1-V3 and III relative to the QRS amplitude. (B) Schematic representation of the rightward, inferiorly, and anterior orientation of the ischemic vector in acute right ventricular strain (T_1_T_2_T_3_/_III_). (C) Representative electrocardiogram of Wellens syndrome demonstrating T-wave inversion in the precordial and lateral leads without involvement of the inferior leads. (D) Computed tomography showing a markedly dilated right ventricle (I) and confirming bilateral pulmonary embolism (II).

Although anterior T-wave inversion is commonly associated with acute coronary syndrome, the distribution in this case suggests an alternative mechanism. T-wave inversion is most pronounced in leads oriented toward the right side of the heart, specifically V1-V3 and lead III (Figure 2A), indicating a rightward and inferior repolarization vector.

In acute PE, right ventricular (RV) pressure overload leads to ischemia and repolarization abnormalities. Common electrocardiographic findings include sinus tachycardia, the classic S_I_Q_III_T_III_ pattern, right bundle branch block, and T-wave inversion in V1-V4,^1^ occasionally extending to inferior leads, a finding that has been associated with adverse prognosis.^2^ Kosuge et al^3^ showed that T-wave inversion maximal in V1-V2 accompanied by negativity in leads V1 and III strongly favors PE over anterior acute coronary syndrome.

Given the anterior and rightward position of the RV, the resulting ischemic vector is directed anteriorly, rightward, and inferiorly, producing predominant T-wave inversion in leads V1-V3 and III. We propose the T_1_T_2_T_3_/_III_ pattern (Figure 2B) as a descriptive mnemonic to facilitate recognition of this characteristic distribution of repolarization abnormalities in PE, analogous to the classic S_I_Q_III_T_III_ pattern.

This pattern may help distinguish PE from other causes of T-wave inversion. Wellens syndrome,^4^ which occurs after resolution of chest pain due to critical proximal left anterior descending artery stenosis, typically presents with deep or biphasic T-wave inversion in leads V2-V3, sometimes extending to the remaining precordial and lateral leads, but usually without inferior involvement (Figure 2C). Takotsubo cardiomyopathy typically produces more diffuse and symmetric T-wave inversion, often accompanied by QT prolongation, and should be considered in the appropriate clinical context. In contrast, apical hypertrophic cardiomyopathy is characterized by increased QRS voltages and giant negative T waves predominantly affecting the left precordial leads.

Imaging revealed a markedly dilated and dysfunctional RV (Figure 2D(I)), and computed tomography pulmonary angiography confirmed bilateral PE (Figure 2D(II)). Given RV dysfunction and clinical severity, mechanical thrombectomy was performed because a recent ischemic stroke represented a relative contraindication to thrombolysis, resulting in rapid clinical improvement.

In summary, T-wave inversion in both anterior and inferior leads—particularly when maximal in V1-V3 and III (the T_1_T_2_T_3_/_III_ pattern)—is a useful electrocardiographic mnemonic clue that may facilitate recognition of PE with RV involvement.

## Funding Support and Author Disclosures

The authors have reported that they have no relationships relevant to the contents of this paper to disclose.
